# Mutator dynamics in sexual and asexual experimental populations of yeast

**DOI:** 10.1186/1471-2148-11-158

**Published:** 2011-06-07

**Authors:** Yevgeniy Raynes, Matthew R Gazzara, Paul D Sniegowski

**Affiliations:** 1 Department of Biology, University of Pennsylvania, Philadelphia, PA 19104-6018, USA

## Abstract

**Background:**

In asexual populations, mutators may be expected to hitchhike with associated beneficial mutations. In sexual populations, recombination is predicted to erode such associations, inhibiting mutator hitchhiking. To investigate the effect of recombination on mutators experimentally, we compared the frequency dynamics of a mutator allele (*msh2*Δ) in sexual and asexual populations of *Saccharomyces cerevisiae*.

**Results:**

Mutator strains increased in frequency at the expense of wild-type strains in all asexual diploid populations, with some approaching fixation in 150 generations of propagation. Over the same period of time, mutators declined toward loss in all corresponding sexual diploid populations as well as in haploid populations propagated asexually.

**Conclusions:**

We report the first experimental investigation of mutator dynamics in sexual populations. We show that a strong mutator quickly declines in sexual populations while hitchhiking to high frequency in asexual diploid populations, as predicted by theory. We also show that the *msh2Δ *mutator has a high and immediate realized cost that is alone sufficient to explain its decline in sexual populations. We postulate that this cost is indirect; namely, that it is due to a very high rate of recessive lethal or strongly deleterious mutation. However, we cannot rule out the possibility that *msh2*Δ also has unknown directly deleterious effects on fitness, and that these effects may differ between haploid asexual and sexual populations. Despite these reservations, our results prompt us to speculate that the short-term cost of highly deleterious recessive mutations can be as important as recombination in preventing mutator hitchhiking in sexual populations.

## Background

Ever since a seminal paper on mutation rate evolution by A. H. Sturtevant [1], two modes of selection on the genomic mutation rate have been recognized: **i**) indirect selection resulting from associations between mutation-rate-modifying alleles at certain loci and fitness-modifying alleles at other loci, and **ii**) direct selection resulting from the fitness effects of mutation-rate-modifying alleles themselves. In the absence of significant evidence for direct selection, most theoretical studies of mutation rate evolution have focused on the indirect selection experienced by mutation rate modifiers and its contrasting effects in sexual and asexual populations [reviewed in [2]].

It has long been argued that because the majority of new fitness-affecting mutations are expected to be deleterious, there should be continual selection pressure toward a lower mutation rate [1,3]. However, in asexual populations, modifiers that increase the genomic mutation rate (mutators) can rise in frequency if associated with beneficial mutations, and a substantial experimental literature in support of such mutator hitchhiking has developed [4-8]. In contrast, in sexual populations recombination is expected to erode associations between beneficial mutations and mutator alleles, preventing mutator hitchhiking and leading to mutator decline due to persistent deleterious mutational pressure [[2,9-11], but see [12-15]].

Surprisingly, the fate of mutator alleles in sexual populations has yet to receive experimental attention. Here, we investigate mutator dynamics in sexual and asexual populations of *S. cerevisiae*. The mutator allele (hereafter, *msh2Δ*) was constructed by replacing the coding sequence of *MSH2*, a component of the yeast mismatch repair system [16], with a kanamycin resistance cassette [17] previously shown to have minimal fitness effect [18]. We established sexual and asexual experimental populations initially polymorphic for this mutator allele and followed mutator frequencies in these populations through time. In light of the results, we discuss the nature of selection experienced by *msh2Δ *in these populations.

## Results

### Mutator dynamics in asexual populations

**Figure 1:**
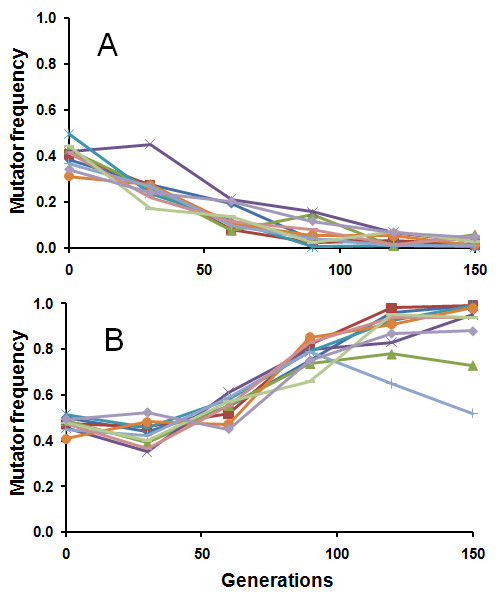
**Mutator dynamics in asexual populations**. Mutator strains decline toward extinction in all ten haploid populations **(A)**. Mutator strains hitchhike to higher frequencies in all ten of the diploid populations, approaching fixation in seven of them **(B)**.

Mutator-carrying strains declined in all haploid asexual populations, approaching extinction in approximately 150 generations (Figure 1A). In contrast, mutator strains rose in frequency at the expense of wild-type strains in all diploid asexual populations after a short initial lag (Figure 1B); furthermore, the mutators appeared to approach fixation in seven of the ten diploid asexual populations by 150 generations of propagation. In asexual populations, the combined strength of both direct and indirect selection acting on the mutator can be very roughly estimated from the change in mutator frequency during the course of the experimental propagation [[19], p193]. Selection coefficients estimated in this way were *s_hap _= *0.031 ± 0.004 (S.E.M.), against the mutator in haploid populations, and *s_dip _*= 0.023 ± 0.002 (S.E.M.) in favor of the mutator in diploid populations. Importantly, these selection coefficients were calculated based on deviations from the initial mutator-to-wild type ratios and not on the dynamics of the actual fitness-affecting mutations that arose in the two subpopulations: therefore, they provide only a very crude measure of the total selection strength acting on mutators. Below, we address how these selection coefficients may nevertheless allow for inferences about the nature of selection experienced by *msh2Δ *in our populations.

### Mutator dynamics in sexual populations

**Figure 2:**
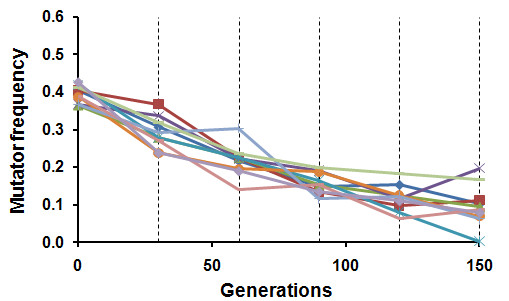
**Mutator dynamics in sexual populations**. The mutator allele declines in frequency in all sexual diploid populations. Dashed vertical lines indicate the times when sporulation was induced and yeast cells were allowed to mate randomly.

In all populations propagated with sexual reproduction, the frequency of the *msh2Δ *allele declined from about 50% towards loss over the 150 generations of propagation (Figure 2). Estimating the strength of selection against *msh2Δ *based on its rate of decline depends on the assumed degree of dominance of its fitness effect. Assuming that the deleterious effect of the *msh2Δ *allele is recessive (see Discussion), we can estimate the strength of selection against it over the full duration of propagation of the sexual populations as *s_rec _*= 0.070 ± 0.009 (S.E.M.) [[19], p192].

### Competitive fitnesses of mutator and wild-type strains before and after propagation

As measured in short-term competition assays (see Methods), the fitness of the haploid mutator strain (YPS3460) relative to that of the wild-type strain (YPS3343) was 0.976 ± 0.002 (S.E.M) before propagation, which is significantly different from one (*t_9 _*= 5.84, two-tailed *p *< 0.01). The fitness of the diploid mutator strain (YPS3484) relative to the wild-type strain (YPS3485) was 1.005 ± 0.001 (S.E.M) before propagation; the difference from one is marginally non-significant (*t_9 _*= 1.974, two-tailed *p *= 0.08). In diploid asexual populations, the relative fitness of mutators increased significantly (*F*_1,24 _= 192.3, *p *<< 0.001) during the experimental propagation, rising to approximately 1.09 based on assays of three different mutator clones isolated at generation 180. In haploid populations, two of three assayed mutator clones showed a significant (*F*_1,19 _= 26.39, *p *<< 0.001) increase in relative fitness, rising to approximately 1.025 in the 160 generations of propagation; the third mutator clone had a relative fitness of 0.964 which was statistically indistinguishable from 0.976 (*t_14 _*= 1.385, two-tailed *p *= 0.19).

## Discussion

### Mutator dynamics and the predicted qualitative effect of recombination

In line with previous studies demonstrating mutator hitchhiking [4,7,20,21], we observed episodes of substantial mutator increase in all diploid asexual populations. Direct competitions between the ancestral diploid mutator and wild-type strains revealed no fitness advantage that could account for the mutator rise in the experimental populations. To assess whether sweeps of beneficial mutations that could facilitate mutator hitchhiking occurred in the diploid asexual populations, we also measured relative fitnesses of mutator clones isolated after experimental propagation. The evolved mutator clones were substantially and significantly more fit than the ancestral mutator strain, strongly suggesting that mutators rose in frequency by hitchhiking with beneficial mutations.

In sexual populations, the *msh2Δ *mutator allele never experienced bouts of hitchhiking similar to those observed in asexual populations, instead declining almost uniformly toward loss. Given the evidence of hitchhiking and fitness evolution in our diploid asexual populations as well as previously published reports of rapid adaptation and high beneficial mutation rates in experimental populations of diploid yeast [21-25], the failure of *msh2Δ *to hitchhike in sexual populations is consistent with the predicted qualitative effect of recombination in separating mutators from beneficial mutations [9,10].

However, the swift decline of *msh2Δ *in these populations also reveals a strong cost of the mutator allele in the sexual populations that was not observed in the asexual diploid populations. This realized cost of *msh2Δ *in the sexual populations (based on its rate of decline) was greater than its realized benefit in the asexual diploid populations and was alone sufficient to explain mutator decline in the sexual populations. Importantly, our diploid asexual and sexual populations differed in more than the presence of meiotic recombination. Sexual diploid populations produced haploid spores, while asexual diploid populations never went through a haploid stage.

### The realized cost of *msh2Δ*

In an effort to distinguish between potential direct costs of the *msh2Δ *allele and an indirect cost due to an increased influx of deleterious mutations, we compared relative fitnesses of ancestral wild-type and mutator strains in both diploid and haploid populations before and after propagation. In diploids, the fitnesses of the two strains were statistically indistinguishable before propagation. In haploids, the mutator strain was slightly less fit than the wild-type strain before propagation, and the difference was consistent with the observed decline of *msh2Δ *during the propagation experiment. These results could be interpreted as supporting a direct fitness cost of *msh2Δ *applying only in haploids. Alternatively, given that the majority of strongly deleterious mutations are recessive [26], indirect selection against *msh2Δ *could account for our observation that mutators declined only in populations with a haploid growth stage (sexual populations and asexual haploid populations). Such an indirect cost of *msh2Δ *would be consistent with previous studies that have inferred a high rate of recessive deleterious mutation in *msh2Δ*-carrying strains [21,27-31]. An indirect cost of *msh2Δ *operating mostly through recessive strongly deleterious mutations could also explain why the mutators were able to spread in our asexual diploid populations, as also previously observed by Thompson *et al*. [21]. In our experiments, fitness measurements of haploid mutators isolated after propagation showed that two out of three clones increased in fitness significantly, suggesting that mutator decline in these populations was not due to gradual accumulation of mildly deleterious mutations; however, an immediate cost of *msh2Δ *could have been a consequence of highly deleterious or lethal mutations, which do not accumulate in populations.

The nature of any direct fitness effects of *msh2Δ *that could explain our results is somewhat unclear. In addition to its role in mismatch repair [16,32], wild-type *MSH2 *has been shown to prevent homeologous recombination and associated chromosomal rearrangements [33-35] and to remove nonhomologous DNA during genetic recombination initiated by double-strand breaks [36-38]. These findings hint at a possible meiosis-specific direct fitness effect of *msh2Δ*. Consistent with this notion is the finding of Reenan and Kolodner [28] that not all spore mortality in *msh2Δ *homozygotes can be accounted for by recessive deleterious mutations. It is worth noting, however, that a meiosis-specific effect of *msh2Δ *could only have contributed to the fitness cost of the mutator in our sexual populations and would not account for the mutator decline in our asexual haploid populations.

### Implications

Whether the realized cost of *msh2*Δ in our sexual populations was direct or indirect, our results have some implications for both theoretical studies of mutator dynamics and the understanding of mutation rate evolution in natural populations. Most studies of mutation rate evolution have adopted the simplifying assumption that mutator alleles are themselves neutral in the short term. Obviously, if a mutator allele were directly deleterious as a consequence of some pleiotropic effect, it would be intrinsically disfavored in both asexual and sexual populations. Notably, mismatch repair alleles that are involved in meiotic recombination may be particularly likely to have a direct effect on fitness in addition to their effect on mutation rate.

On the other hand, if we are correct in positing that the cost of *msh2*Δ is primarily indirect rather than direct, then our results suggest that, similarly to a direct cost, an indirect cost of a mutator allele may apply immediately in some situations. If strongly deleterious mutations are rare, then indirect selection against a mutator in an asexual population will develop only gradually after a shift in mutation rate [39-42]. However, if strongly deleterious mutations are common, then indirect selection against a mutator can act over the short term to inhibit mutator hitchhiking in situations where recessive deleterious mutations are expressed.

The notion that recombination is perhaps not the only factor that can prevent mutator hitchhiking in sexual populations has implications for mutation rate evolution in natural populations that can reproduce sexually but go through a prominent haploid phase: for example, fungi, algae and some plants. Consistent with effective selection against higher mutation rates, there are no reports to date of mutator strains at substantial frequencies in natural yeast populations, despite the low rates of outcrossing inferred in such populations [43,44]. While mutation rate data are scarce in plants, it seems likely that mutators could also carry a high and immediate indirect cost in plant species with a prominent gametophyte phase (mosses, ferns, and others). In contrast, haploid stage selection against mutator alleles should be weaker in populations where haploids are less important and act primarily to transport genetic material, as in multicellular animals. To our knowledge, this idea that the efficacy of selection against higher mutation rates may depend on population life cycle was first suggested by Sturtevant [1].

Current estimates of genomic deleterious mutation rates (*U_d_*) range from a low value of 0.0002 in the bacterium *E. coli *[45] to potentially above 1 in many sexually reproducing species [14]. In light of studies demonstrating mutator hitchhiking in experimental bacterial populations [5,7,8,46,47] it may seem surprising that genomic mutation rates in natural bacterial populations have not evolved to higher levels. One possible explanation for low genomic mutations rates in bacteria is that mutator hitchhiking is inhibited in haploid bacterial populations by the associated deleterious load. Another potential explanation, however, is the prevalence of horizontal gene transfer [48,49], which could act similarly to sexual recombination in preventing mutator hitchhiking. Currently available evidence suggests that horizontal gene transfer in bacteria is rather widespread, as it might have accounted for anywhere between 1.6 - 32.6% of some bacterial genomes [50]. Interestingly, despite regular recombination, most multicellular eukaryotes have strikingly high genomic mutation rates, potentially suggesting a role for nonadaptive forces in shaping mutation rates in these species [15].

Assuming that selection acting on *msh2Δ *in our haploid yeast populations was primarily indirect, we can roughly estimate the minimum deleterious mutation rate in yeast as *U_d _*= 5.5 × 10^-4 ^(see Methods). Current published estimates of *U_d _*in *S. cerevisiae *range from 4.8 × 10^-5 ^to 1.1 × 10^-3 ^[24,30,51]; the deleterious mutation rate in our populations was probably closer to the high end of this range [30], because the indirect cost of *msh2Δ *in our populations would have been reduced somewhat by the mutator's association with new beneficial mutations. Moreover, the high percentage of haploid lethal mutations demonstrated in [30] is consistent with the observed immediate fitness effect of *msh2Δ *in our experiments.

## Conclusions

Our study raises the potential importance of haploid phase selection and high deleterious mutation rates on mutator success in sexually reproducing populations. We suggest that the interaction of recombination with these factors is an important area for further theoretical and empirical research. We have shown that a mutator allele of *msh2 *declines rapidly in frequency in experimental populations of sexually reproducing yeast while nearly hitchhiking to fixation in isogenic asexual populations. The realized cost of the *msh2Δ *allele could be due to an increase in strongly deleterious and lethal mutations, and it could also be due to an unknown direct effect that operates only in haploids and sexually reproducing populations. Whatever its cause, the magnitude of the fitness effect of *msh2Δ *is sufficient to obscure the expected qualitative effect of recombination on mutator dynamics. Further research will be necessary to unambiguously demonstrate the theoretically expected effect of recombination on mutation rate evolution in sexual populations.

## Methods

### Strains and Media

All experiments were conducted with two isogenic haploid *S. cerevisiae *strains, YPS3329 (ho::nat, MSH2, MAT α) and YPS3343 (ho::nat, MSH2, MAT a), previously isolated from Mettler's Woods, NJ [52] or their derivatives. The msh2::kan allele (generously provided by Clifford Zeyl), a complete deletion of the coding sequence of the mismatch repair locus *MSH2*, was used to replace wild-type *MSH2 *in YPS3343 using a standard transformation protocol [53], producing isogenic strain YPS3460. The kanamycin cassette confers dominant resistance to the drug G418 which was used as a selective agent to assess mutator frequencies in the evolution experiments. Fluctuation tests [54] indicated that the *msh2Δ *allele provided for a 60-fold increase in the haploid mutation rate over wild-type *MSH2 *at the *URA3 *locus (see additional file 1 for fluctuation test procedure). Diploid homozygous mutator (YPS3484) and wild-type (YPS3485) strains were isolated from the mating of strains YPS3329 and YPS3460 and confirmed by tetrad analysis. Low-glucose YPD (0.2% glucose) and sporulation media were prepared according to standard recipes [55].

### Experimental propagation

Sets of replicate populations were established with mutator alleles at starting frequencies of roughly 50%. Each population was propagated in 10 ml of low-glucose YPD broth at 30°C with shaking at 200 rpm and with 1:1000 daily dilution to fresh medium. Given the daily population bottleneck of 10^5 ^cells and the number of generations between transfers (≈10), the effective population size was approximately 10^6 ^[56]. The asexual haploid populations were established by combining aliquots of roughly 5 × 10^4 ^cells from overnight mutator (YPS3460) and wild-type (YPS3343) cultures into replicate flasks. The asexual diploid populations were established by combining aliquots of roughly 5 × 10^4 ^cells from overnight diploid mutator (YPS3484) and diploid wild-type (YPS3485) cultures into replicate flasks. To establish the sexual populations, haploid wild-type and mutator strains of opposite mating type (strains YPS3329 and YPS3460) were mated and sporulated. Then approximately 10^5 ^cells of the resulting culture were aliquoted into replicate flasks to start sexual populations.

Growth medium was replaced with sporulation medium after every three days of serial passage for the sexual populations, and the populations were allowed to sporulate for three days without serial passage. Sporulated cultures were washed, treated with beta-mercaptoethanol to kill unsporulated cells, and digested with zymolyase. The resulting haploid spores were mass-mated on low-glucose YPD plates, and serial transfer was restarted by inoculating fresh low-glucose YPD broth with about 10^5 ^cells from the mated cultures. Sporulation efficiency was estimated at about 70% by direct observation under a compound microscope, and thus the sexual populations are likely to have had roughly the same effective size as the asexual populations.

In both sexual and asexual populations, mutator frequencies were assessed by replica plating 100 to 300 randomly sampled colonies, derived from each population at each assayed time point, from YPD plates to YPD plates containing 200 μg/ml of G418. Allele frequencies in the diploid sexual populations were assessed immediately after mass mating by assuming Hardy-Weinberg genotypic proportions.

### Relative fitness assays

Relative fitnesses of mutator and wild-type strains were assayed in two-day direct competitions. Ancestral strains were inoculated from frozen stocks into 10 ml of low-glucose YPD. Evolved mutator clones were isolated on low-glucose YPD agar plates and inoculated into 10 ml of low-glucose YPD. After growing to stationary densities, the two competitors were then diluted 2000-fold and introduced into replicate flasks of low-glucose YPD. The cultures were grown overnight and transferred into fresh low-glucose YPD medium after 1:1000 dilution. Culture samples were plated on YPD agar at the beginning and at the end of the 2^nd ^day of propagation (after ≈ 20 generations). Mutator frequencies were estimated by replica plating about 200 colonies per competition onto YPD+ G418 agar plates. Relative - fitnesses were inferred from the shift in mutator frequencies using standard population genetics theory [19].

### Deleterious mutation rate estimation

The coefficient of indirect selection against a mutator subpopulation at equilibrium deleterious mutational load is predicted to be approximately *mU_d _*[39,57], where *m *is the factor by which the mutator elevates the genomic deleterious mutation rate and *U_d _*is the genomic deleterious mutation rate in the wild-type [2]. Considering that we have estimated *m *to be approximately 60 and assuming that increase in *U_d _*is proportional to increase in the mutation rate assayed, we can approximate the deleterious mutation rate in our populations as *U_d _*= *s_hap_/m *= 5.5 × 10^-4^. The validity of this estimate depends on whether the realized cost of the mutator was exclusively indirect and, as implied above, on whether the populations had attained equilibrium mutational load.

## Authors' contributions

YR and PDS designed the experiments. YR and MRG carried out the propagation experiments and fluctuation assays. YR and PDS wrote the manuscript, which all authors read and approved.

## Supplementary Material

Additional file 1 **Mutation rate estimation**. Description of the fluctuation test method used to estimate mutation rates. Click here for file
