# Supplementary material for: Mutator dynamics in sexual and asexual experimental populations of yeast
Source: BMC Evol Biol. 2011 Jun 7;11:158. doi: 10.1186/1471-2148-11-158 (PMC3141426; doi:10.1186/1471-2148-11-158)
Supplement: Additional file 1 — Mutation rate estimation. Description of the fluctuation test method used to estimate mutation rates. [file 1471-2148-11-158-S1.DOC]

## Mutation rate estimation

Modified fluctuation assays [1, 2] were performed to measure mutation rates of the experimental strains. Yeast strains were first inoculated from frozen stock into 10ml of YPD medium and grown overnight. After a serial dilution, a portion of the overnight cultures (about 1000 cells) was then transferred into 10ml of fresh YPD and the resultant cultures were grown for two days. A set of ten replicate populations for each fluctuation test was then set up by inoculating 30 ml of YPD with approximately 100 cells each. After two days of growth 300μl (for wild-type) and 30μl of 1:10 dilution (for mutator) of each culture was centrifuged, resuspended in 100μl of sterile water and plated on minimal agar plates [3] containing 1mg/ml 5-fluoro-orotic acid (5FOA) to select for mutants [4]. In addition, samples of five of the ten cultures were plated on permissive YPD agar after appropriate serial dilutions to estimate total population size. Mutant counts from 5FOA and permissive YPD plates were used to estimate mutation rates with “Mutation Rate Calculator” software (P. J. Gerrish, personal communication), based on the method presented in [2].

**References**

1. Luria SE, Delbrück M: **Mutations of bacteria from virus sensitivity to virus resistance.** *Genetics* 1943, **28:**491-511.

2. Gerrish P: **A simple formula for obtaining markedly improved mutation rate estimates.** *Genetics* 2008, **180:**1773-1778.

3. Rose MD, Winston F, Hieter P: *Methods in Yeast Genetics: A Laboratory Course Manual* Cold Spring Harbor: Cold Spring Harbor Laboratory Press; 1990.

4. Zeyl C, de Visser JAGM: **Estimates of the rate and distribution of fitness effects of spontaneous mutation in *Saccharomyces cerevisiae*.** *Genetics* 2001, **157:**53-61.
